# Phytochemical Composition and Antioxidant Activity of *Portulaca oleracea*: Influence of the Steaming Cooking Process

**DOI:** 10.3390/foods10010094

**Published:** 2021-01-05

**Authors:** María del Pilar Fernández-Poyatos, Eulogio J. Llorent-Martínez, Antonio Ruiz-Medina

**Affiliations:** Department of Physical and Analytical Chemistry, Faculty of Experimental Sciences, University of Jaén, Campus Las Lagunillas, E-23071 Jaén, Spain; mpoyatos@ujaen.es (M.d.P.F.-P.); ellorent@ujaen.es (E.J.L.-M.)

**Keywords:** *Portulaca oleracea*, purslane, oleracein, alkaloid, antioxidant, phytochemical, HPLC-MS

## Abstract

In this work, we compared the phenolic composition and antioxidant capacity of methanolic extracts of raw and steamed aerial parts of *Portulaca oleracea* L. Two new cyclo-dopa amides were identified, named oleraceins X and Y, along with six known ones (oleraceins A, B, C, N, J, and U). Compounds identification and quantification were done by high-performance liquid chromatography with diode array and mass spectrometry detections. The most abundant compounds were phenolic alkaloids (oleraceins), and the main quantified compounds were isocitric and citric acids, with concentrations of 500–550 and 440–600 mg/100 g dried extract, respectively. The study of both the influence of the steaming process in *Portulaca oleracea* L. and total phenolic content and radical scavenging assays (ABTS^·+^ and DPPH) were also carried out. The total individual phenolic content of raw *Portulaca* decreased from 1380 mg/100 g DE to 1140 mg/100 g DE after the steaming process. The antioxidant capacity in ABTS and DPPH assays decreased approximately 50 and 40%, respectively, after samples were cooked by steaming. The raw extracts presented the highest concentration of bioactive compounds, as well as higher antioxidant and radical scavenging values.

## 1. Introduction

Vegetables are sources of antioxidants, minerals, and vitamins, and their consumption can provide a balanced and healthy diet. They are associated with the prevention and reduction of cancer, cardiovascular and other chronic diseases. Phytochemical compounds such as phenolics are some of the contributors to these health benefits [1]. When studying the potential health benefits of foods, including vegetables, it is important to evaluate not only the composition of raw food, but also that of cooked foods, including complex food dishes [2,3].

**Figure 1:**
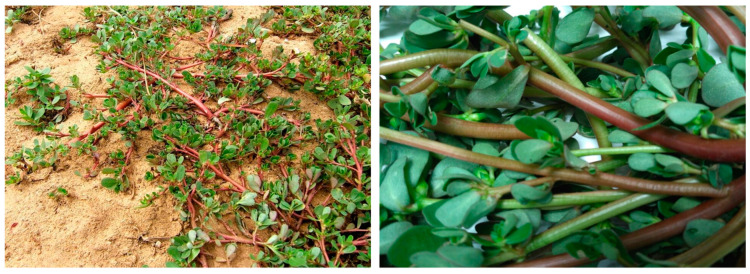
*Portulaca oleracea* L.

*Portulaca oleracea* L., commonly called purslane, belongs to the genus *Portulaca* from the family *Portulacaceae*. Purslane is a well-known edible annual herb that grows in Europe, Africa, North America, Australia, and Asia, considering it as an invasive species by their widespread distribution [4]. It grows in orchards, gardens, crop fields, even in roadsides. They are succulent, up to 30 cm high. The plant exhibits small leaves, with oval shape and dark green color; its stems are reddish and have many small yellow flowers during May to September in which the interior contains tiny black seeds [5] (Figure 1).

With regards to its health benefits, *Portulaca oleracea* has been called “vegetable for long life” in the Chinese medicinal culture of plants [6], and it is used in folk and traditional medicine as a remedy for many ailments, including dermatitis, abdominal pain, headache, inflammation, intestinal worms, high fever, urinary tract infections, etc., due to possessing a large spectrum of pharmacological effects (analgesic, antioxidant, anti-inflammatory, bactericide, hypocholesterolemic, and hypoglycemic) [7,8,9]. This plant also contains a high level of proteins, sterols, carotenoids, and polysaccharides [10,11,12]. It is an important source of omega-3 fatty acids [13,14]. Moreover, it has a wide range of vitamins (A, C, E, and some of complex-B) [10,12] and minerals (Ca, Fe, Mn, P, and Se) [12,15]. In addition, the determination of volatile compounds, amino acids, antimicrobial assays [10], as well as tocopherols, organic acids, and cytotoxicity tests [11] have been carried out in studies of *Portulaca oleracea*.

Purslane is rich in polyphenols, which includes phenolic acids and flavonoids. Several reports on raw *Portulaca oleracea* have been made about its phenolic composition, total phenolic, and total flavonoid contents. Compounds such as caffeic acid, ferulic acid, kaempferol, quercetin, and rutin have been previously identified [10,11,12,13,14,16,17,18]. This plant also shows a class of alkaloids, which are secondary metabolites, named cyclo-dopa amides, or oleraceins, are increasing the general interest [12,13,19]. These compounds show more or similar potent antioxidant characteristics than some natural antioxidants such as vitamin C and vitamin E [12,13].

*Portulaca oleracea* is consumed both raw and cooked. It is an important component of green salad and its leaves and stems are used raw, alone, or with other vegetables. Fresh juices are also prepared, and purslane is commonly added to soups, steamed, in teas. or used as potherb [5,10,12,13,20]. The impact of cooking treatment on vegetables can cause changes in nutritional composition and levels of bioactive compounds [21]. Studies on the effects on cooking have been carried out in *Portulaca oleracea*, observing differences according to the type of cooking, but mainly focus on boiling and bleaching, or cooked with some food additives such as lemon juice or vinegar [22,23,24].

This work aimed to determine the composition of the phenolic content of raw and steamed aerial parts of *Portulaca oleracea*, as well as its antioxidant activity, thus evaluating the effect of the steaming cooking process, which has not been reported so far. In addition, the structure of two new oleraceins has been elucidated. The hypothesis was that the steaming process would decrease both phenolic composition and antioxidant activity when compared to raw *Portulaca* samples, but still maintaining a reasonable number of bioactive compounds.

## 2. Materials and Methods

### 2.1. Chemicals and Reagents

All reagents and standards were of analytical reagent grade. Standards of caffeic acid, catechin, citric acid, ferulic acid, hydroxytyrosol, kaempferol, quercetin, and sinapic acid were purchased from Sigma-Aldrich (Madrid, Spain). All solutions were prepared in methanol (MeOH) HPLC-grade (Sigma-Aldrich). LC-MS grade acetonitrile (Panreac; Barcelona, Spain) and ultrapure water (Milli-Q Waters purification system; Millipore; Milford, MA, USA) were also used.

Ethanol (96%), Folin-Ciocalteu′s phenol reagent (FCR) and sodium carbonate (Na_2_CO_3_) were purchased from Panreac (Madrid, Spain). 2,2′-azinobis (3-ethylbenzthiazoline-6-sulfonic acid) (ABTS; ≥98%), 2,2-diphenyl-1-picrylhydrazyl (DPPH; 95%), gallic acid monohydrate (>98%), 6-hydroxy-2,5,7,8-tetramethylchroman-2-carboxylic acid (Trolox; 97%), formic acid (98–100%), and potassium persulfate (>99%) were obtained from Sigma-Aldrich.

### 2.2. Sample Preparation and Extraction

Portions of fresh aerial parts of different *Portulaca oleracea* plants were randomly and carefully collected, approximately 1000 g, in an orchard in Andújar (southeast of Spain; 38°03′40.7” N 4°02′58.4” W, 235 m a.s.l.), in October 2019. Botanical authentication was carried out by the botanist Dr. Carlos Salazar Mendías (Department of Animal Biology, Plant Biology and Ecology of the University of Jaén, Spain). Only the freshest samples were selected. They were mixed into the same pool, washed with Milli-Q water, and cut into small portions (approximately 10-cm-long). A portion of fresh (raw) *Portulaca oleracea* was analyzed to check the initial phenolic content and antioxidant capacity, whereas another portion was steamed. The last one was cooked in a traditional stainless-steel steamer (three pieces: A pot, a steamer basket, and a lid) for 5 min. Cooking time was selected according to the usual time used in domestic recipes.

Previous works compared different extracting solvents (methanol, ethanol, ethyl acetate, and water) for the analysis of *Portulaca* composition, reporting that methanol was the most suitable one for the extraction of phenolic compounds [25,26,27]. Hence, we selected MeOH for the extractions of phenolics from raw and steamed samples.

**Figure 2:**
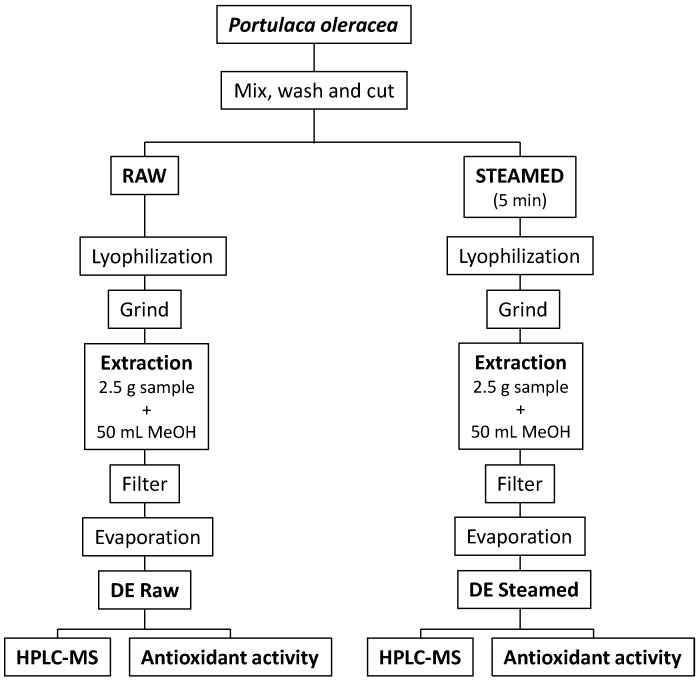
Scheme of the experiments. DE: Dried extract.

For both raw and steamed samples, aerial parts were lyophilized (Lyoquest -55 ECO, Telstar; Barcelona, Spain) and crushed with a grinder. Ultrasound-assisted extraction was done by placing 2.5 g of dry material in 50 mL MeOH for 10 min (Qsonica Sonicators; Newton, CT, USA) with a power of 55 W and a frequency of 20 kHz (50% power). Each sample was extracted in triplicate. Then, solutions were filtered through Whatman No.1 filters and the solvent was evaporated under reduced pressure in a rotary evaporator at 40 °C. Dried extracts (DE) were stored at −20 °C until analysis. A scheme of the process is shown in Figure 2.

### 2.3. HPLC Analysis

The raw and steamed extracts were analyzed by high-performance liquid chromatography with diode-array and mass spectrometry detection in an Agilent Series 1100 with a G1315B diode array detector and an ion trap mass spectrometer (Esquire 6000, Bruker Daltonics) with an electrospray interface. Detail conditions are given in [28]. We also used an Agilent 1200 HPLC equipped with an Agilent 6530B quadrupole-time-of-flight mass spectrometer (Q-TOF MS) to confirm the identity of the compounds by exact mass determination.

Calibration curves for caffeic acid, catechin, citric acid, ferulic acid, hydroxytyrosol, kaempferol, quercetin, and sinapic acid at concentrations 1–100 µg mL^−1^ in MeOH. Chromatograms were recorded at 280 nm for catechin and hydroxytyrosol; 320 nm for caffeic acid, ferulic acid, and sinapic acid; and 350 nm for kaempferol and quercetin. For citric acid, the instrument was operated in product ion scan MS/MS mode with the selected MS/MS transition 191→111, and voltage amplitude of 0.6 V.

### 2.4. Assay for Total Phenolic Content and Determination of Antioxidant Capacity

For the following analyses, the procedures were adapted from [29]. Total phenolic content (TPC) and antioxidant assays were carried out using a UV-Vis Spectrophotometer (Zuzi Spectrophotometer, model 4201/50; Beriain, Navarra, Spain). For TPC, 3–5 mg mL^−1^ of dried extracts were analyzed, and the obtained results are reported as mg of gallic acid equivalents (GAE) per g of dried extract. Regarding the antioxidant capacity, two different spectrophotometric radicals scavenging tests (ABTS^·+^ and DPPH). Sample solutions of 0.15 mg mL^−1^ and 1 mg mL^−1^ in MeOH were prepared for ABTS^+^ and DPPH assays, respectively. Results are expressed as µmol of Trolox equivalents (TE) per g of dried extract, for both assays. All assays were done in triplicate.

## 3. Results and Discussion

In this work, we compared the composition of the aerial parts of *Portulaca oleracea* in its raw and steamed form. The most common cooking methods for this plant are boiling and steaming. Previous studies on other vegetables, such as pumpkin, reported steaming as better than boiling regarding antioxidant activity and phenolic content [30]; similarly, a study performed on cauliflower reported that due to the degree of exposure to heat and water (different wet-thermal process cooking), steaming induced the least reductive effect on phytochemical components [31]. We thus studied the effect of steaming on the antioxidant and phenolic composition of *Portulaca oleracea*.

### 3.1. HPLC-MS Analysis of Raw and Steamed Extracts

**Figure 3:**
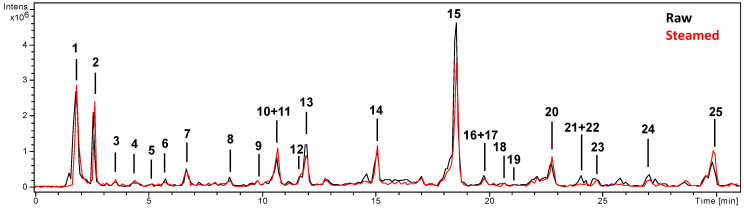
HPLC-ESI/MS^n^ base peak chromatograms (BPC) of the raw and steamed extracts of *Portulaca oleracea* aerial parts.

Compounds characterization was carried out by mass spectrometry, using both negative and positive ion modes, although most of the compounds were identified in negative mode (Table 1). The base peak chromatograms of raw and steamed extracts of *Portulaca oleracea* aerial parts are shown in Figure 3. We identified or tentatively characterized 24 compounds; almost 30% were phenolic acids and 25% flavonoids. There was a higher percentage (33%) that corresponded with alkaloid compounds, specifically oleraceins.

#### 3.1.1. Phenolic Acids

Compound **4** displayed deprotonated molecular ion at *m/z* 371 and fragment ions *m/z* 353, 209, 191, and 129. This fragmentation pattern has been reported in the bibliography for both caffeoylglucaric acid and hydroxyferulic acid-hexoside. Hence, Q-TOF was used to calculate the exact mass, and the experimental fragmentation pattern was compared with the database METLIN. Compound **4** was thus unequivocally identified as caffeoylglucaric acid. In the same way, compounds **5** and **7** were identified as caffeic acid glucuronide isomers.

Compounds **9** and **17** were ferulic acid derivatives. Compound **9**, with [M-H]^−^ at *m/z* 355, suffered the neutral loss of a hexoside (162 Da) yielding the MS^2^ base peak at *m/z* 193 and the fragment ions characteristic of ferulic acid at *m/z* 178, 149 and 134; this compound was characterized as ferulic acid-*O*-hexoside [32]. Compound **17** exhibited [M-H]^−^ ion at *m/z* 309 and was tentatively characterized as ferulic acid derivative due to the fragment ions at *m/z* 193, 149 and 134 (typical of ferulic acid).

Compound **6** was identified as caffeic acid-*O*-hexoside. It presented [M-H]^−^ ion at *m/z* 341, produced a fragment ion at *m/z* 179 (caffeic acid) due to the neutral loss of a hexoside, and typical caffeic acid fragments at *m/z* 161 and 135 [29]. Compound **11** showed [M-H]^−^ ion at *m/z* 385. This compound was characterized as a hydroxycinnamoyl glycoside, namely sinapic acid-*O*-hexoside or sinapoyl hexoside. The base peak fragment ion in MS^2^ was at *m/z* 223 (neutral loss of 162 Da, corresponding to a hexoside molecule). Other fragment ions were at *m/z* 205 (loss of water, 18 Da), *m/z* 179 (loss of 44 Da, carbon dioxide), and *m/z* 149 (30 Da, generated by the losses of two CH_3_ radicals) [33].

#### 3.1.2. Flavonoids

Compounds **8** and **12** displayed [M-H]^−^ at *m/z* 289 and identical fragmentation patterns (*m/z* 245, 205, 203, and 179). These compounds were identified as catechin and epicatechin, respectively, considering that epicatechin elutes after catechin in reversed-phase column [34].

Compounds **18** and **24** exhibited [M-H]^−^ ions at *m/z* 463 and suffered the neutral loss of 162 Da (hexoside), producing quercetin at *m/z* 301 (characteristic fragments at *m/z* 179 and 151), so both compounds were identified as quercetin-*O*-hexoside isomers [32].

Compound **22** was named as kaempferol-*O*-hexoside. It showed [M-H]^−^ ion at *m/z* 447 and the neutral loss of 162 Da yielded the aglycone kaempferol at *m/z* 285/284 [35].

Compound **23** was characterized as isorhamnetin-*O*-hexoside, presenting the aglycone at *m/z* 315, due to the neutral loss of a hexose molecule, and typical fragment ion of isorhamnetin at *m/z* 300 [34].

#### 3.1.3. Alkaloids

**Figure 4:**
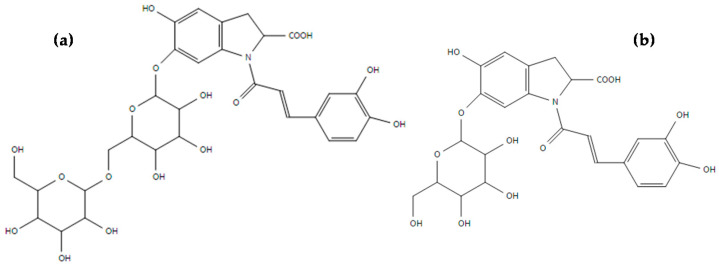
Proposed structures of oleracein X (**a**) and oleracein Y (**b**).

Compounds **10**, **13**, **14**, **15**, **16**, **19**, **20,** and **21** were characterized as cyclo-dopa amides, named oleraceins. In previous works, the presence of these phenolic alkaloids has been reported in *Portulaca oleracea* [19,36,37]. Compound **10** was identified as oleracein C, with [M-H]^−^ at *m/z* 664 and fragment ions at *m/z* 502 ([M-Glc-H]^−^; Glc = glucoside) and 340 ([M-Glc-Glc-H]^−^) [19]. Compound **13** showed an [M-H]^−^ ion at *m/z* 680. This compound has not been previously identified and, according to the study of the fragments, we propose a similar structure to oleracein J but without the ferulic acid group [M-C_10_H_8_O_3_-H]^−^ [19], naming it as oleracein X, following the order of nomenclature of the compounds of this alkaloid family, whose structure was determined as 5-hydroxy-1-caffeic acyl-2,3dihydro-1*H*-indole-2-carboxylic acid-6-*O*-diglucoside (C_30_H_35_NO_17_) (Figure 4). The fragment ions of oleracein X were *m/z* 518 (neutral loss of 163 Da corresponding to a caffeic acyl group ([M-C_9_H_6_O_3_-H]^−^)), *m/z* 356 (neutral loss of 325 Da belonging to two glucoside molecules ([M-C_12_H_20_O_10_-H]^−^)), *m/z* 246 (neutral loss of 435 Da corresponding to 325 Da and 110 Da from two glucoside (Glc) molecules and a 1,2-dihydroxybenzene molecule ([M-2Glc-C_6_H_5_O_2_-H]^−^)), and *m/z* 202 (neutral loss of 479 Da belonging to 435 Da of the loss explained previously and an additional loss of 44 Da from a carboxylic group ([M-2Glc-C_6_H_5_O_2_-CO_2_-H]^−^)). The proposed molecular formula for compound **13** (oleracein X) has been confirmed by exact mass using Q-TOF analysis.

Compound **14** showed an [M-H]^−^ ion at *m/z* 518 and fragment ions at *m/z* 356 (neutral loss of 163 Da corresponding to a glucoside molecule ([M-Glc-H]^−^)), *m/z* 246 (neutral loss of 110 Da from a 1,2-dihydroxybenzene molecule ([M-Glc-C_6_H_5_O_2_-H]^−^)) and *m/z* 202 (neutral loss of 44 Da from the previous fragment ion due a carboxylic group ([M-Glc-C_6_H_5_O_2_-CO_2_-H]^−^)). This compound has not been previously described and, according to the fragmentation pattern, we propose a new compound called oleracein Y, whose structure was determined as 5-hydroxy-1-caffeic acyl-2,3dihydro-1*H*-indole-2-carboxylic acid-6-*O*-glucoside (C_24_H_25_NO_12)_ (Figure 4), similar to oleracein V structure but without a hydroxyl group (C_24_H_25_NO_13_) [12]. The molecular formula proposed for compound **14** (oleracein Y) was also confirmed by exact mass using Q-TOF results.

**Figure 5:**
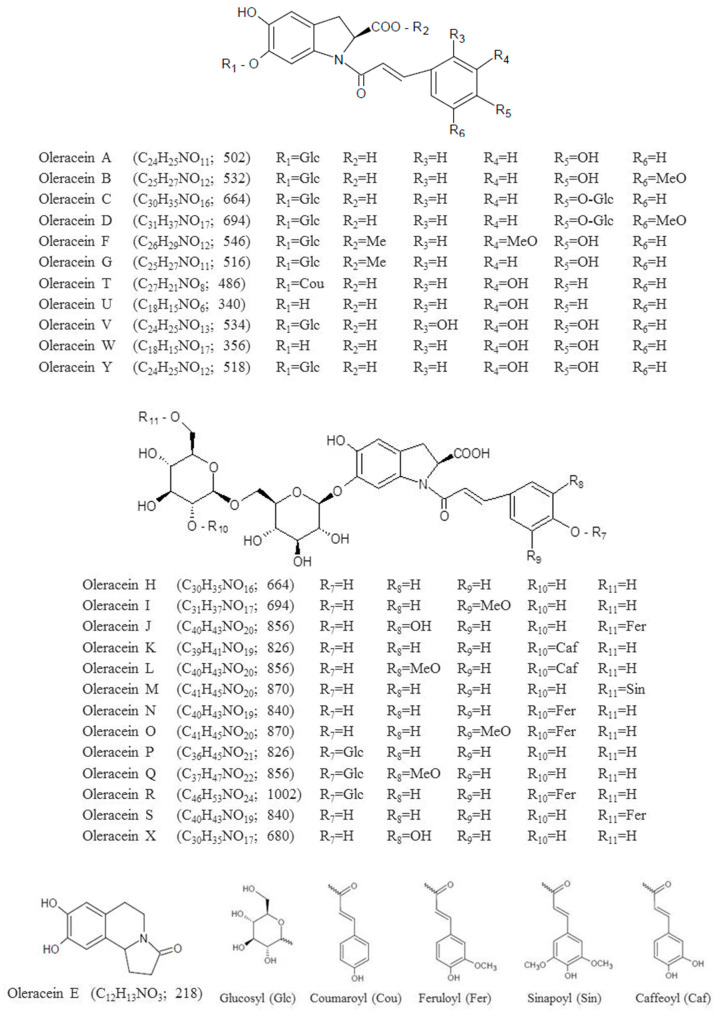
Chemical structures and molecular ions [M-H]^−^ of oleraceins A-Y.

Compound **15** was the highest peak in the chromatograms of both raw and steamed extracts. It showed [M-H]^−^ ion at *m/z* 502 and the characteristic fragmentation pattern of oleracein A (*m/z* 340, 252, 296) [10]. Compound **16** was identified as oleracein B. It presented an [M-H]^−^ ion at *m/z* 532 and the typical fragmentation of this alkaloid (*m/z* 370, 326, 282) [10]. Compound **19** was characterized as another cyclo-dopa amide, specifically, oleracein N. It displayed an [M-H]^−^ ion at *m/z* 840 and the fragment ions characteristics of this alkaloid (*m/z* 694, 664, 518) [19]. Compound **20** was tentatively characterized as oleracein U, due to the [M + H]^+^ ion at *m/z* 342 and base peak at *m/z* 147, previously reported in bibliography [13]. Compound **21** was identified as oleracein J. It showed [M-H]^−^ ion at *m/z* 856 and the typical fragments ion of this cyclo-dopa amide (*m/z* 694, 680, 356, and 246) [19]. In Figure 5, all the oleraceins reported in scientific literature (oleracein A to oleracein W, including the new oleraceins X and Y) have been gathered, showing the chemical structures and molecular ions [M-H]^−^ [12,19,38,39,40].

#### 3.1.4. Other Compounds

Compounds **1** and **2** were organic acids, showing the same deprotonated molecular ions. They were characterized as isocitric acid and citric acid, respectively, by comparison with an analytical standard of citric acid. Compound **3**, with [M-H]^−^ at *m/z* 315, and fragment ions at *m/z* 153 and 123, was named as hydroxytyrosol hexoside [34].

### 3.2. Quantification of Phenolic Compounds in All Extracts

Fifteen compounds were quantified in the analyzed extracts of *Portulaca oleracea* by HPLC, measuring phenolic acids at 320 nm, flavonoids at 350 nm, and epicatechin and hydroxytyrosol hexoside at 280 nm. Citric acid was quantified in MS/MS mode with an analytical standard (Table 2).

**Figure 6:**
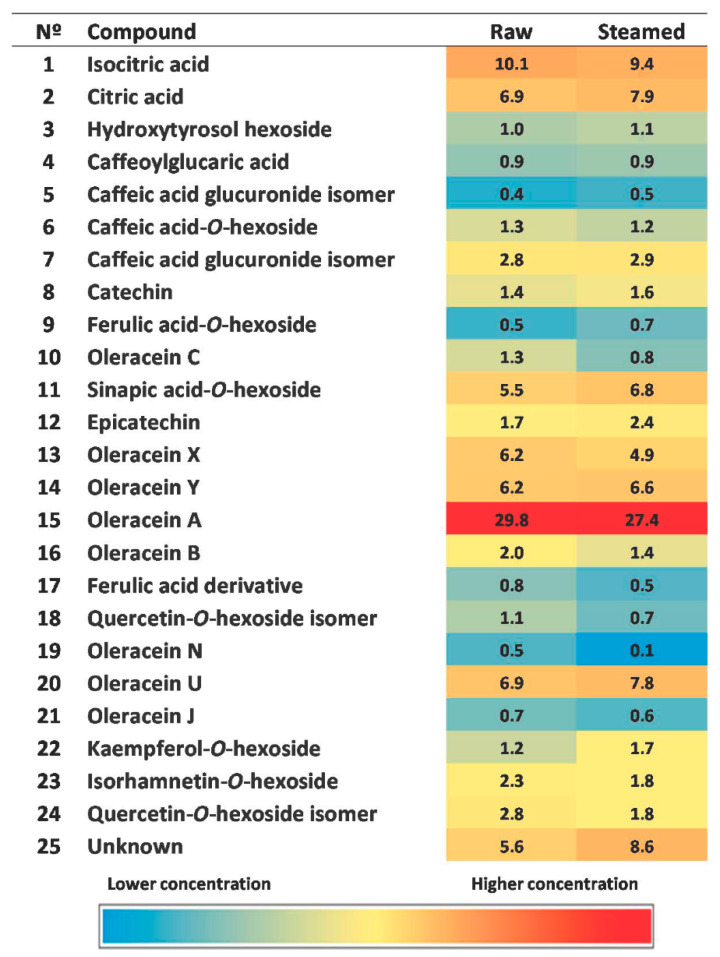
Relative peak areas and heat map of the raw and steamed extracts of *Portulaca oleracea* aerial parts.

Oleraceins were not quantified because there are no commercial standards of these phenolic alkaloids. We made a heatmap with the values of the areas (%) of each of the identified compounds with respect to total area, in order to visualize the contribution of each of the compounds (Figure 6) to the extracts. Areas were calculated for each compound using Extracted Ion Chromatograms at the corresponding deprotonated molecular ion. This figure shows that oleracein A is the major compound in both extracts, followed by isocitric acid, citric acid, oleracein U, sinapic acid-*O*-hexoside and the new oleraceins X and Y. In previous works, oleracein A also appears as the major compound in *Portulaca oleracea* [13,19].

From a qualitative point of view, raw and steamed extracts presented the same phenolic profile, although higher concentrations of phenolic acids, flavonoids and other compounds were observed in raw *Portulaca*, being TIPC (Total Individual Phenolic Content, defined as the sum of all individual phenolic concentrations) values of 1380 mg/100 g DE and 1140 mg/100 g DE in raw and steamed samples, respectively. It was thus observed the initial hypothesis of a decrease in bioactive compounds during the steaming process was confirmed. However, the decrease in TIPC was lower than 20%. The losses of each individual compound after the steaming process are given in Table 2, observing that the concentration of most compounds, except epicatechin, decreased approximately between 10 and 20%.

Isocitric acid and citric acid were the most abundant compounds in all extracts, presenting values of 550 and 600 mg/100 g DE for raw extract, and 500 and 440 mg/100 g DE for steamed extract. Both compounds represented in raw and steamed extracts more than 80% of the TIPC. Citric acid has been reported in *Portulaca oleracea* in the range of 1655– mg/100 g DE [41]. Citric acid is commonly used in the food, pharmaceutical, and cosmetic industries. In addition to being a flavor enhancing additive, this compound has been reported to behave as an antioxidant [42].

Sinapic acid-*O*-hexoside was the phenolic acid found in greater quantity, reaching values of 38 mg/100 g DE and 32 mg/100 g DE in raw and steamed extracts, respectively, which meant 27% of the total phenolic acids in both cases. A value of 22.1 mg/100 g DE was previously reported in literature for this plant [11].

Aerial parts of *Portulaca oleracea* were also rich in flavonoids such as those derived from kaempferol, quercetin, and epicatechin, the latter being the only one of all quantified phenols that had a higher value in the steamed extract (30 mg/100 g DE) than in the raw extract (28 mg/100 g DE), which respectively represented 37% and 31% of the total flavonoids. In previous works, flavonoids (derivatives of kaempferol and quercetin, among others) were also reported in *P. oleracea* [12,13,43].

### 3.3. Total Phenolic Content and Antioxidant Activity

Spectrophotometric methods are still frequently used to determine total phenolic content, as well as to report the antioxidant activity of plant extracts. In our opinion, their simplicity, low-cost, and possibility to compare with previous research makes them very useful. However, it is important to consider that the data obtained by these assays are preliminary (mainly *screening* methods), and the use of HPLC-MS (or similar techniques) is mandatory for the identification and quantitation of phytochemicals. Each antioxidant assay presents a different mechanism and specific handicaps that need to be taking into account for a proper interpretation of the results [44].

**Figure 7:**
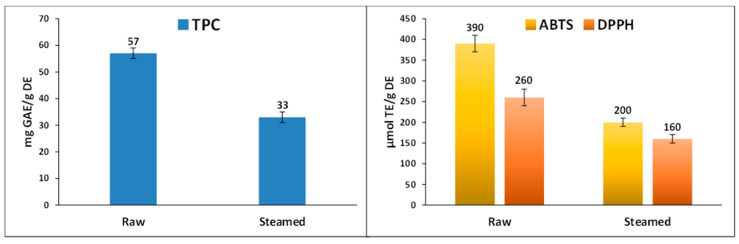
Total phenolic content (TPC) and antioxidant assays (ABTS and DPPH) of raw and steamed *Portulaca oleracea* aerial parts. Values are given as mean ± standard deviation of three parallel measurements. GAE: Gallic acid equivalent; TE: Trolox equivalent; DE: Dried extract.

The results of the TPC assay for raw and steamed *Portulaca oleracea* extracts are given in Figure 7. In accordance with HPLC-MS results, the raw extract possessed a higher concentration of phenolic content (57 mg GAE/g DE) than the steamed extract (33 mg GAE/g DE), showing a statistically significant difference between their values. In previous literature, total bioactive components for raw *Portulaca oleracea* have been reported, with maximum values between 3.6 and 13.4 mg GAE/g DE [14,18,25,26,27]. In a previous work comparing the raw, boiled, and blanched *P. oleracea*, it was observed that the raw sample had a higher TPC value (23 mg GAE/g DE) compared to the values of the cooked samples (boiled 19 mg GAE/ g DE and blanched 10 mg GAE/g DE) [23]. In another study, Naser Aldeen et al. obtained slightly higher values for cooked than raw purslane (13.13 mg GAE/g DE and 12.75 mg GAE/g DE, respectively) [24].

The antioxidant capacity of *Portulaca oleracea* extracts was also evaluated; results are summarized in Figure 7. The study of the antioxidant capacity of plant extracts is of great interest in order to provide new and safer natural antioxidants for pharmaceutical and food industries. So, in this work, free radical quenching (ABTS^+^ and DPPH assays) was studied to examine the antioxidant effects of raw and steamed *Portulaca oleracea* extracts. In both assays, the antioxidant effects showed a similar trend to total bioactive compounds, decreasing after the steaming process, in agreement with the initial hypothesis of the work. The values for ABTS^·+^ and DPPH assays of the raw extract were 390 µmol TE/g DE and 260 µmol TE/g DE, and for the steamed extract were 200 µmol TE/g DE and 160 µmol TE/g DE, so the raw extract was more active on free radicals than the steamed extract. However, it can be observed that a higher decrease was observed in ABTS values with respect to DPPH ones. A previously reported value in an ABTS assay for raw purslane was 102.7 µmol TE/g DE, lower than that obtained in our study [43]. In another work on aerial parts of *Portulaca oleracea*, the cooking process with lemon juice as additive increased the antioxidant activity compared to raw samples in the DPPH assay [24]. The decrease in antioxidant activity observed after the steaming process is in agreement with the decrease in phenolic content (Table 2). Considering that the decrease of concentration was similar in all compounds (as well as TIPC), the lower antioxidant activity in steamed samples cannot be attributed to particular compounds. Although the observed antioxidant capacity (in both raw and steamed samples) was mainly due to the most abundant compounds, the whole extract must be considered to account for the antioxidant potency.

## 4. Conclusions

In this work, we have reported the phenolic profile and antioxidant activity of aerial parts of *Portulaca oleracea* L. The most abundant compounds were oleraceins, followed by citric and isocitric acids. In addition, the influence of the steaming cooking process has been reported for the first time. The initial hypothesis of a decrease in both the concentration of bioactive compounds and the antioxidant capacity was confirmed by HPL-MS (quantification of phytochemicals) and antioxidant assays; the decrease in phenolic concentration and antioxidant activity after the steaming process were in agreement. However, it is important to mention that the steamed samples still contained a significant amount of the initial compounds and kept a considerable antioxidant activity. We also identified two new oleraceins that have not been previously reported in scientific literature. Their structures were elucidated by ion-trap mass spectrometry and confirmed by exact mass using Q-TOF.

## Figures and Tables

**Table 1:** Characterization of phytochemicals found in extracts raw and steamed of *Portulaca oleracea* by HPLC–DAD/ESI–MS^n^.

No.	t_R_ (min)	[M-H]^−^ *m*/*z*	*m*/*z* (% Base Peak)	Assigned Identification
1	1.8	191	MS^2^ [191]: 173 (41), 111 (100)	Isocitric acid
2	2.6	191	MS^2^ [191]: 173 (80), 111 (100)	Citric acid *
3	3.6	315	MS^2^ [315]: 153 (100), 135 (52) MS^3^ [315→153]: 123 (100)	Hydroxytyrosol hexoside
4	4.4	371	MS^2^ [371]: 353 (18), 209 (100), 191 (44) MS^3^ [371→209]: 191 (100) MS^4^ [371→209→191]: 129 (100)	Caffeoylglucaric acid
5	5.2	355	MS^2^ [355]: 209 (29), 191 (100) MS^3^ [355→191]: 149 (94), 129 (100)	Caffeic acid glucuronide isomer
6	5.8	341	MS^2^ [341]: 179 (100), 161 (23), 135 (22) MS^3^ [341→179]: 135 (100)	Caffeic acid-O-hexoside
7	6.7	355	MS^2^ [355]: 337 (11), 209 (34), 191 (100), 129 (4)	Caffeic acid glucuronide isomer
8	8.6	289	MS^2^ [289]: 245 (100), 205 (25), 203 (12), 179 (17) MS^3^ [289→245]: 203 (100), 175 (45), 159 (41), 123 (50)	Catechin *
9	9.8	355	MS^2^ [355]: 193 (100), 178 (9), 134 (12) MS^3^ [355→193]: 178 (100), 149 (42)	Ferulic acid-O-hexoside
10	10.4	664	MS^2^ [664]: 502 (100) MS^3^ [664→502]: 340 (100)	Oleracein C
11	10.7	385	MS^2^ [385]: 247 (45), 223 (100), 205 (57) MS^3^ [385→223]: 208 (20), 179 (13), 164 (100), 149 (13) MS^4^ [385→223→164]: 149 (100)	Sinapic acid-O-hexoside
12	11.6	289	MS^2^ [289]: 245 (100), 205 (45), 203 (22), 179 (18) MS^3^ [289→245]: 217 (89), 203 (100), 161 (55)	Epicatechin
13	11.9	680	MS^2^ [680]: 518 (100), 356 (59), 246 (28), 202 (18) MS^3^ [680→518]: 356 (100), 246 (37), 202 (14) MS^4^ [680→518→356]: 246 (100), 202 (32)	Oleracein X
14	15.1	518	MS^2^ [518]: 356 (100), 246 (25), 202 (8) MS^3^ [518→356]: 246 (100), 202 (28), 150 (10) MS^4^ [518→356→246]: 202 (100)	Oleracein Y
15	18.5	502	MS^2^ [502]: 340 (100), 252 (13), 145 (3) MS^3^ [502→340]: 296 (65), 252 (100), 194 (47), 145 (87)	Oleracein A
16	19.7	532	MS^2^ [532]: 370 (100), 326 (6), 282 (9), 175 (4) MS^3^ [532→370]: 282 (100), 161 (72), 175 (63)	Oleracein B
17	19.7	309	MS^2^ [309]: 193 (100), 134 (5) MS^3^ [309→193]: 149 (100), 134 (80)	Ferulic acid derivative
18	20.5	463	MS^2^ [463]: 301 (100), 271 (5), 179 (4), 151 (4) MS^3^ [463→301]: 271 (100), 255 (43), 229 (51), 179 (96), 151 (43)	Quercetin-O-hexoside isomer
19	21.1	840	MS^2^ [840]: 694 (100), 664 (77), 518 (61) MS^3^ [840→664]: 518 (100), 340 (58)	Oleracein N
20	22.7	342 (+)	MS^2^ [342]: 177 (22), 147 (100), 119 (14) MS^3^ [342→147]: 119 (100)	Oleracein U
21	24.0	856	MS^2^ [856]: 694 (100), 680 (16), 356 (35), 246 (14) MS^3^ [856→694]: 356 (100), 246 (81)	Oleracein J
22	24.0	447	MS^2^ [447]: 285 (100), 284 (76), 255 (45) MS^3^ [447→284]: 257 (24), 255 (100), 227 (37)	Kaempferol-O-hexoside
23	24.5	477	MS^2^ [477]: 357 (11), 315 (100), 285 (31), 271 (19), 151 (8) MS^3^ [477→315]: 300 (27), 285 (100), 271 (69) MS^4^ [477→314→285]: 271 (100)	Isorhamnetin-O-hexoside
24	27.0	463	MS^2^ [463]: 301 (100), 179 (3), 151 (2) MS^3^ [463→301]: 179 (100), 151 (41) MS^4^ [463→301→179]: 151 (100)	Quercetin-O-hexoside isomer
25	29.8	677	MS^2^ [677]: 659 (42), 645 (100), 627 (30), 617 (64), 585 (22) MS^3^ [677→645]: 627 (80), 541 (73), 489 (100), 462 (78) MS^4^ [677→645→489]: 462 (49), 445 (53), 417 (100)	Unknown

* Identified with analytical standards.

**Table 2:** Quantification of compounds in raw and steamed extracts of *Portulaca oleracea*.

*N*°.		Raw	Steamed	Loss (%)
*Phenolic Acids*				
4	Caffeoylglucaric acid	18.1 ± 0.6 ^a^	16.4 ± 0.7 ^b^	9.4 ± 0.9
5	Caffeic acid glucuronide isomer	14.30 ± 0.08 ^a^	12.3 ± 0.4 ^b^	14 ± 2
6	Caffeic acid-O-hexoside	14.12 ± 0.08 ^a^	12.9 ± 0.7 ^b^	9 ± 4
7	Caffeic acid glucuronide isomer	27.8 ± 0.7 ^a^	23 ± 1 ^b^	17 ± 2
9	Ferulic acid-O-hexoside	9.3 ± 0.5 ^a^	7.7 ± 0.2 ^b^	17 ± 2
11	Sinapic acid-O-hexoside	38.0 ± 0.4 ^a^	32 ± 1 ^b^	16 ± 2
17	Ferulic acid derivative	18.44 ± 0.03 ^a^	15 ± 1 ^b^	19 ± 5
Total		140 ± 1 ^a^	119 ± 2 ^b^	15 ± 1
*Flavonoids*				
12	Epicatechin	28 ± 2 ^a^	30 ± 3 ^a^	---
18	Quercetin-O-hexoside isomer	13.2 ± 0.3 ^a^	11.2 ± 0.7 ^b^	15 ± 3
22	Kaempferol-O-hexoside	15 ± 1 ^a^	13.6 ± 0.9 ^a^	9.0 ± 0.1
23	Isorhamnetin-O-hexoside	15.9 ± 0.2 ^a^	13 ± 1 ^b^	18 ± 5
24	Quercetin-O-hexoside isomer	18.4 ± 0.7 ^a^	13.8 ± 0.7 ^b^	25 ± 1
Total		91 ± 2 ^a^	82 ± 3 ^b^	10 ± 1
*Other compounds*				
1	Isocitric acid	550 ± 40 ^a^	500 ± 40 ^a^	9.1 ± 0.7
2	Citric acid	600 ± 50 ^a^	440 ± 20 ^b^	26 ± 3
3	Hydroxytyrosol hexoside	3.89 ± 0.02 ^a^	3.83 ± 0.08 ^a^	2 ± 1
Total		1150 ± 60 ^a^	940 ± 40 ^b^	18.2 ± 0.8
TIPC		1380 ± 60 ^a^	1140 ± 40 ^b^	17.4 ± 0.7

Values (mg/100 g DE) are mean ± SD of three parallel measurements. Different superscripts (^a^ and ^b^) indicate significant differences in the extracts (*p*< 0.05).

## Data Availability

Data available on request.
